# Physical activity and exercise in dementia: clinical relevance and emerging insights

**DOI:** 10.3389/frdem.2026.1843904

**Published:** 2026-07-23

**Authors:** Thyparambil Aravindakshan Pramodkumar, Viswanathan Mohan

**Affiliations:** 1 Department of Research Operations and Diabetes Complications, Madras Diabetes Research Foundation, Chennai, India; 2 Chairman, Madras Diabetes Research Foundation (ICMR-Collaborating Centre of Excellence) and Dr Mohan’s Diabetes Specialities Centre (IDF Centre of Excellence in Diabetes), Chennai, India

**Keywords:** cognitive, dementia, diabetes, exercise, physical activity, therapies

## Abstract

Dementia continues to pose a growing challenge worldwide, particularly in aging populations where effective disease-modifying treatments remain limited. In this context, attention has increasingly shifted toward non-pharmacological approaches, including physical activity and structured exercise. Evidence accumulated over the past decade suggests that regular exercise may offer modest but meaningful benefits across several domains. These include improvements in cognitive function—especially executive function and attention—as well as gains in mobility, daily functioning, and psychological well-being. While the extent of cognitive improvement varies across studies, a consistent pattern emerges indicating greater benefit when interventions are initiated earlier in the disease course. Beyond clinical outcomes, several biological mechanisms have been proposed, including enhanced cerebral perfusion, upregulation of neurotrophic factors such as brain-derived neurotrophic factor, and attenuation of neuroinflammatory processes. At the same time, challenges remain. Differences in exercise protocols, issues related to adherence, and variability in patient populations make it difficult to define an optimal approach. Despite these limitations, physical activity represents a practical, low-cost strategy that can be integrated into routine care. Further research is needed to refine intervention design and understand long-term effects.

## 1 Introduction

Dementia is a progressive condition marked not only by cognitive decline but also by functional impairment and behavioral changes that significantly affect quality of life (Cipriani et al., 2020). Globally, an estimated 55 million people are currently living with dementia, and this number is projected to rise to over 139 million by 2050, driven largely by population aging and increasing life expectancy (World Health Organization, 2021). As life expectancy increases, the burden of dementia is expected to rise sharply, particularly in low- and middle-income settings where healthcare resources are often limited (Mattap et al., 2022). Although pharmacological therapies have advanced over the years, their impact remains largely symptomatic, with little effect on slowing disease progression. This has led to growing interest in lifestyle-based and non-pharmacological interventions (Castellano-Tejedor, 2022). Among these, physical activity has received considerable attention. Observational studies have consistently shown that individuals who remain physically active tend to have a lower risk of developing cognitive impairment and other non-communicable diseases (Yamasaki, 2023; Ford et al., 2021). Therapeutic exercise has been shown to slow physical and cognitive decline, and recent interventional studies suggest it may also benefit individuals already living with dementia. However, its sustained impact on functional outcomes and the persistence of benefits after discontinuation remain unclear (Maria Del Rosario et al., 2026).

The rationale for this approach is supported by a range of biological and clinical observations. Exercise has been linked to improved vascular health, enhanced neuroplasticity, and reductions in systemic inflammation—all of which may play a role in neurodegenerative processes (Ben Ezzdine et al., 2025). However, translating these mechanisms into consistent clinical benefit has proven complex. To provide a clearer conceptual framework, the following section first defines the key terms used throughout this review before discussing the various exercise modalities, their biological mechanisms, clinical evidence, and practical applications in dementia care.

## 2 Physical activity and exercise: definitions and exercise modalities

### 2.1 Key definitions

*Dementia* is a progressive neurodegenerative syndrome characterized by deterioration in memory, executive function, language, attention, and other cognitive domains, resulting in impairment of daily functioning and loss of independence. Alzheimer’s disease is the most common cause of dementia, although vascular dementia, dementia with Lewy bodies, and frontotemporal dementia also contribute substantially to the global disease burden (Hugo and Ganguli, 2014).

*Cognition* encompasses the mental processes involved in learning, memory, attention, language, executive functioning, processing speed, and visuospatial abilities. Progressive impairment in these domains forms the clinical hallmark of dementia and is closely associated with functional decline and reduced quality of life (Hugo and Ganguli, 2014).

*Physical activity* refers to any bodily movement produced by skeletal muscles that increases energy expenditure above resting levels, whereas exercise is a planned, structured, and repetitive form of physical activity performed with the objective of improving or maintaining physical fitness. In dementia research, exercise interventions commonly include aerobic, resistance, balance, flexibility, and multicomponent training, each targeting distinct physiological and cognitive outcomes (Caspersen et al., 1985).

### 2.2 Types of physical activity and exercise

Physical activity includes a range of structured and unstructured movements, varying in intensity and purpose. In clinical and research settings, exercise interventions are generally categorized into aerobic, resistance, balance, and multicomponent training, each contributing differently to overall health and functional outcomes (De la Rosa et al., 2020). Aerobic exercise is the most commonly studied modality and typically includes activities such as walking, swimming, cycling, or low-impact group exercises (Cheng et al., 2025). These activities primarily target cardiovascular fitness and have been associated with improvements in cerebral blood flow and overall brain health (Tomoto et al., 2023). Walking-based interventions, in particular, are widely used due to their simplicity and feasibility in older populations (Brach and Vanswearingen, 2013). Resistance training is a highly effective intervention to counter age-related declines in muscle mass, strength, and physical function, thereby improving resilience and reducing vulnerability in older adults. Evidence supports its role in enhancing mobility, independence, chronic disease management, psychological well-being, and overall quality of life through well-designed, structured programs (Fragala et al., 2019). In individuals with dementia, resistance training has been linked to improvements in functional performance and may also contribute indirectly to cognitive outcomes through enhanced metabolic health (Li et al., 2025). Balance and flexibility exercises are also important, particularly given the increased risk of falls in this population (Sadaqa et al., 2023). Activities such as stretching, yoga, or tai chi can improve postural stability and coordination, thereby reduce fall risk and support safer mobility (Chen et al., 2023; Subramani et al., 2025). In recent years, multicomponent exercise programs that combine aerobic, strength, and balance training have gained attention (Schneider et al., 2025). These programs are thought to offer broader benefits by targeting multiple physiological systems simultaneously and are often associated with more consistent improvements in both cognitive and functional outcomes. Overall, while no single type of exercise appears universally superior, a combination of different modalities, tailored to individual capabilities and preferences, is likely to provide the greatest benefit. Table 1 presents the exercise modalities and their effects in dementia. Understanding these exercise modalities provides the foundation for examining how physical activity influences different forms of dementia and their underlying pathological mechanism.

**Table 1:** Summary of exercise modalities and their effects in dementia.

Exercise type	Examples	Primary targets	Reported benefits in dementia	Key considerations
Aerobic exercise	Walking, cycling, swimming	Cardiovascular fitness, cerebral blood flow	Modest improvements in global cognition, executive function; improved endurance	Easy to implement; requires monitoring in frail individuals
Resistance training	Weight training, resistance bands	Muscle strength, metabolic health	Improved functional capacity, mobility; indirect cognitive benefits	Needs supervision for safety
Balance and flexibility	Yoga, tai chi, stretching	Postural stability, coordination	Reduced fall risk; improved mobility and confidence	Suitable for frail individuals
Multicomponent exercise	Combination of aerobic + strength + balance	Multiple physiological systems	Most consistent improvements in cognition and function	Requires structured programs
Functional training	Daily activity-based exercises	Activities of daily living	Improved independence and quality of life	Highly practical and adaptable

## 3 Cognition and types of dementia

### 3.1 Cognitive effects

The impact of exercise on cognitive outcomes in dementia has been examined in a number of randomized trials and meta-analyses, though findings are not entirely uniform. In general, small but consistent improvements have been reported in domains such as executive function and attention. Effects on memory are less consistent, with some studies showing benefit and others reporting minimal change. One pattern that appears repeatedly is that individuals in earlier stages of cognitive decline tend to respond better to exercise interventions (Saez de Asteasu and Izquierdo, 2023). This may reflect greater neural reserve or the ability to engage more actively in structured programs. At the same time, differences in study design—including the type, intensity, and duration of exercise—make direct comparisons difficult. Overall, while exercise is unlikely to produce dramatic cognitive improvements, the observed benefits are clinically relevant, particularly when considered alongside its broader effects on physical and psychological health (Mandolesi et al., 2018).

### 3.2 Subtypes of dementia and differential response to exercise

Dementia encompasses a heterogeneous group of disorders, with Alzheimer’s disease and vascular dementia being the most common subtypes, often coexisting as mixed dementia. While many studies evaluate dementia as a single entity, emerging evidence suggests that the effects of physical activity may vary across subtypes. In Alzheimer’s disease, exercise is thought to primarily influence neurodegenerative pathways, including neuroplasticity and synaptic function. Improvements in cognitive domains such as executive function and attention have been reported, although effects on memory are less consistent (Cui et al., 2018). In contrast, vascular dementia is more closely linked to cerebrovascular pathology, and therefore may respond more directly to interventions that improve cardiovascular and metabolic health (Kling et al., 2013). Physical activity, through its effects on blood pressure, insulin sensitivity, endothelial function, and cerebral perfusion, may have a particularly important role in this subgroup.

Mixed dementia, which combines neurodegenerative and vascular components, may benefit from both mechanisms, although evidence remains limited (Sarhan et al., 2025). Overall, these differences highlight the importance of considering the subtype of dementia when interpreting study findings and designing exercise interventions.

## 4 Functional outcomes and metabolic diseases

### 4.1 Functional and psychological outcomes

Beyond cognition, the effects of physical activity in individuals with dementia extend to several aspects of daily functioning and psychological well-being. The Lifestyle Interventions and Independence for Elders (LIFE) Study showed that regular exercise can improve mobility, balance, and overall physical performance (Fielding et al., 2011). These changes are particularly important, as they directly influence an individual’s ability to perform activities of daily living and maintain a degree of independence. Improvements in functional capacity are often accompanied by reductions in the risk of falls, which remain a major concern in older adults with cognitive impairment. Structured exercise programs that incorporate strength and balance training are generally effective in reducing fall risk. However, evidence also suggests that the way exercise is delivered may influence outcomes. For instance, the LiFE study demonstrated that a lifestyle-integrated functional exercise approach significantly reduced fall rates, whereas traditional structured exercise programs showed less consistent benefits (Clemson et al., 2012). There is also growing evidence that physical activity can positively influence neuropsychiatric symptoms (Mendonca et al., 2021). Reductions in depressive symptoms, agitation, and anxiety have been reported across several trials, although the magnitude of these effects varies. Needs assessments significantly increased long-term care service utilization (from 37.3 to 45.5% at 6 months) and were associated with a reduction in moderate-to-severe caregiver burden (Su et al., 2025). Psychological factors such as stress, depression, and anxiety are increasingly recognized as important modifiers of disease outcomes in chronic conditions, including metabolic disorders, and may also contribute to cognitive decline and dementia progression (Vidyulatha et al., 2022). Sleep patterns may also improve with regular physical activity, although this area remains less well studied (Alnawwar et al., 2023). Taken together, these findings suggest that the benefits of exercise in dementia are not limited to cognitive outcomes alone, but extend to broader aspects of health and well-being.

### 4.2 Metabolic health, diabetes, and dementia: the role of physical activity

**Figure 1:**
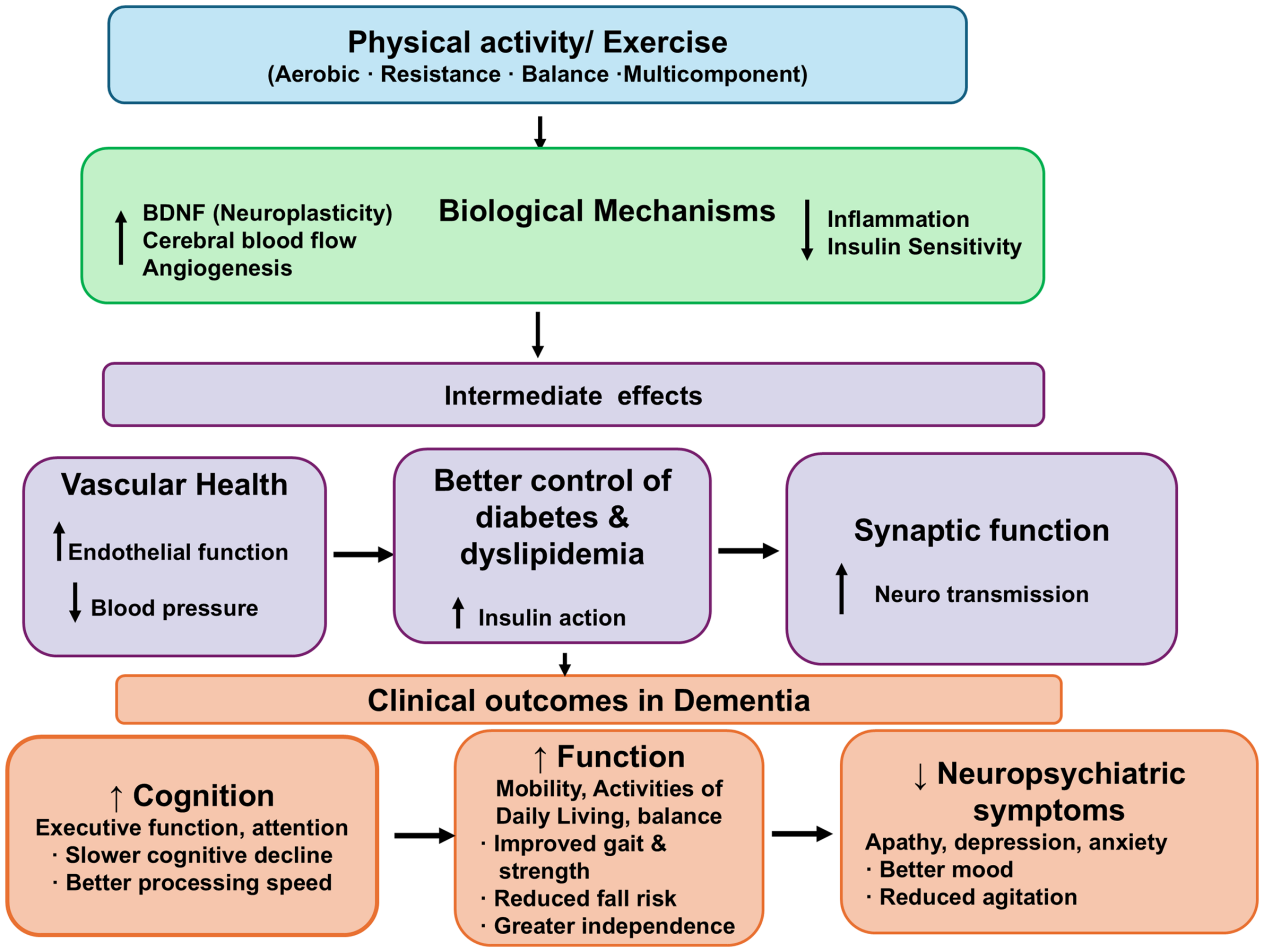
Multidimensional effects of physical activity on cognitive, functional, and metabolic outcomes in dementia.

There is increasing recognition that dementia, particularly Alzheimer’s disease, is closely linked to metabolic dysfunction. Heterogeneity in metabolic disorders, particularly in T2D, suggests varying underlying pathophysiological mechanisms that may differentially influence systemic and neurological outcomes (Mohan et al., 2025). Conditions such as T2D, insulin resistance, obesity, and dyslipidemia have been consistently associated with an increased risk of cognitive decline (Kim and Feldman, 2015). In this context, dementia has even been described by some as a “metabolic disease of the brain or type 3 diabetes” reflecting the role of impaired glucose metabolism and insulin signaling in its pathogenesis (de la Monte and Tong, 2014). Insulin plays an major role in the central nervous system, influencing synaptic plasticity, neuronal survival, and memory processes (Duarte et al., 2012). In states of insulin resistance, these functions may be compromised, contributing to neurodegeneration. In addition, chronic hyperglycemia and associated metabolic disturbances can promote oxidative stress, advanced glycation end-product formation, and vascular damage, all of which may accelerate cognitive decline (Yaffe et al., 2011). These effects are particularly relevant in individuals with or at risk of T2D, where improved metabolic regulation may translate into better cognitive outcomes. Beyond glucose metabolism, exercise also favorably influences lipid profiles, reduces central adiposity, and lowers systemic inflammation (Imierska et al., 2020). Given that vascular contributions to cognitive impairment are common, improvements in cardiometabolic health may play a key role in mediating the benefits of physical activity in dementia. Figure 1 presents the metabolic dysfunction, brain damage, and dementia: a vicious cycle and the protective role of exercise.

Emerging evidence suggests that individuals with coexisting metabolic disorders may derive significant benefit from exercise interventions, although data remain somewhat limited. This highlights the importance of considering metabolic health as an integral component of dementia care, rather than as a separate comorbidity.

## 5 Biological basis of exercise in dementia

### 5.1 Biological mechanisms

**Figure 2:**
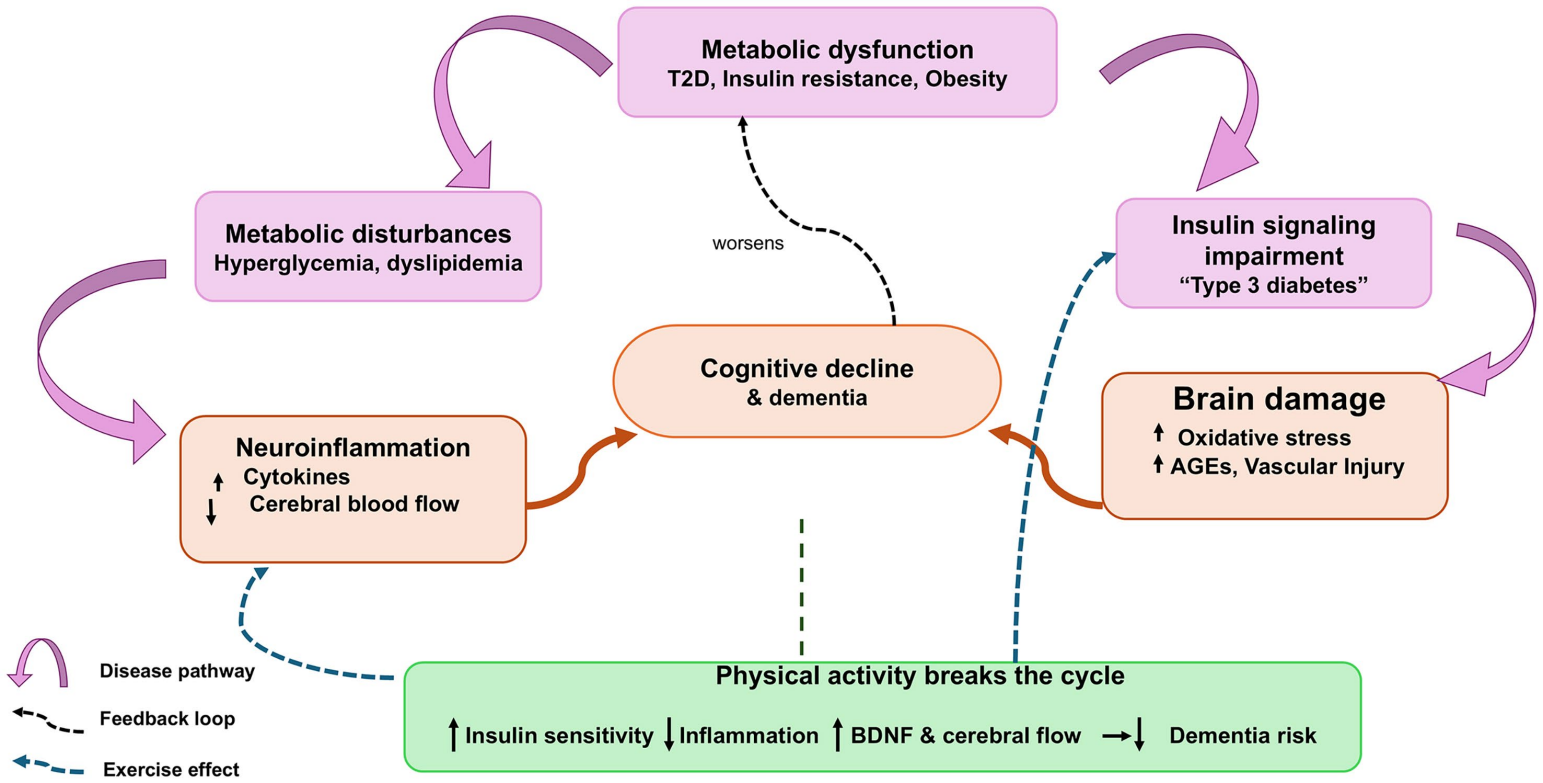
Metabolic dysfunction, brain damage, and dementia: a vicious cycle and the protective role of exercise.

The mechanisms through which physical activity exerts its effects in dementia are complex and likely multifactorial. One of the most widely discussed pathways involves the upregulation of neurotrophic factors, particularly BDNF, which plays a key role in neuronal survival, synaptic plasticity, and learning processes (Colucci-D'Amato et al., 2020). Experimental studies have consistently demonstrated that exercise can increase BDNF levels, although translating these findings into clinical populations remains challenging. In addition to its effects on neuroplasticity, exercise has a well-established role in improving vascular health (de Sousa Fernandes et al., 2020). Enhanced cerebral blood flow and improved endothelial function may contribute to better oxygen and nutrient delivery to the brain, thereby supporting cognitive function. This is particularly relevant given the strong link between vascular risk factors and dementia (Kisler et al., 2017). Microvascular dysfunction, well documented in conditions such as diabetic retinopathy, has also been implicated in the pathogenesis of neurodegenerative diseases including dementia (Pramod Kumar et al., 2023). Another important mechanism involves the modulation of inflammation. Chronic low-grade inflammation has been implicated in the pathogenesis of neurodegenerative diseases, and physical activity may help attenuate this process (Adamu et al., 2024). Similarly, improvements in metabolic health, including better glucose regulation and insulin sensitivity, may indirectly influence brain function, especially in populations with a high burden of metabolic disorders (Gu et al., 2025). It is likely that these mechanisms interact in a collaborative manner, rather than acting independently, contributing to the overall benefits observed with regular physical activity. Figure 2 presents multidimensional effects of physical activity on cognitive, functional, and metabolic outcomes in dementia.

Several mechanisms have been proposed to explain how physical activity might influence cognitive function in dementia. One of the most frequently discussed is the role of neurotrophic factors, particularly brain-derived neurotrophic factor (BDNF), which supports neuronal survival and synaptic plasticity (Bathina and Das, 2015). Exercise has been shown to increase BDNF levels, at least in experimental settings. In addition, physical activity improves cerebral blood flow and vascular function, both of which are closely linked to cognitive health (Sleiman et al., 2016). There is also evidence suggesting that exercise may help reduce chronic low-grade inflammation, which has been implicated in neurodegeneration (Su and Su, 2025). Metabolic factors, including insulin resistance, may also play a role, particularly in populations with a high burden of cardiometabolic disease (Kirk and Klein, 2009; Pramod Kumar and Mohan, 2025; Mohan et al., 1985; Anjana et al., 2023; Mohan et al., 2008). Type 2 diabetes (T2D) may also increase the risk of dementia (Albai et al., 2019). Physical activity has been shown to decreases cardiometabolic risk factors (Mohan et al., 2005). These mechanisms are likely to act in combination rather than isolation, contributing to the overall effect observed in clinical studies. Collectively, these interconnected pathways provide the biological basis for the beneficial effects of exercise in dementia. Given the complexity of these mechanisms, a detailed discussion of the major molecular and physiological pathways—including neuroplasticity and BDNF signaling, other neurotrophic factors, exercise-induced myokines, neuroinflammatory modulation, and vascular-metabolic adaptations—is presented in the following sections.

### 5.2 Neuroplasticity and brain-derived neurotrophic factor (BDNF)

Regular physical activity promotes neuroplasticity through multiple molecular and cellular pathways, among which brain-derived neurotrophic factor (BDNF) is considered one of the most important mediators (Colucci-D'Amato et al., 2020). BDNF is highly expressed in the hippocampus and cerebral cortex, where it regulates neuronal survival, differentiation, synaptic plasticity, and long-term potentiation, processes that are essential for learning and memory (Bathina and Das, 2015; Colucci-D'Amato et al., 2020). Exercise-induced increases in BDNF activate the tropomyosin receptor kinase B (TrkB), triggering intracellular signaling pathways that enhance dendritic spine formation, facilitate hippocampal neurogenesis, strengthen synaptic connectivity, and improve neuronal resilience against age-related degeneration (Sleiman et al., 2016; Colucci-D'Amato et al., 2020). Consequently, regular exercise may enhance learning, memory, and executive function while slowing cognitive decline in individuals with dementia. Experimental and clinical studies consistently demonstrate that aerobic exercise increases circulating and hippocampal BDNF concentrations, although the magnitude of this response varies according to exercise intensity, duration, and disease stage (Cheng et al., 2025; Sleiman et al., 2016; Munoz Ospina and Cadavid-Ruiz, 2024; de Sousa Fernandes et al., 2020).

### 5.3 Other neurotrophic factors supporting brain health

Although BDNF has been the most extensively investigated neurotrophic factor, several additional growth factors contribute to the neuroprotective effects of exercise. Insulin-like growth factor-1 (IGF-1) (Bathina and Das, 2015), vascular endothelial growth factor (VEGF), nerve growth factor (NGF), and glial cell line-derived neurotrophic factor (GDNF) play complementary roles in maintaining neuronal integrity (Calvo et al., 2023). Exercise increases circulating IGF-1, which readily crosses the blood–brain barrier and acts synergistically with BDNF to stimulate hippocampal neurogenesis and synaptic plasticity. Similarly, VEGF promotes angiogenesis and improves cerebral perfusion, thereby enhancing oxygen and nutrient delivery to metabolically active brain regions (Trejo et al., 2001). NGF and GDNF further support neuronal survival and repair by maintaining cholinergic and dopaminergic neuronal function. Collectively, these neurotrophic factors contribute to enhanced neuronal resilience and may partly explain the beneficial effects of long-term exercise on cognitive function.

### 5.4 Exercise-induced myokines and the muscle–brain axis

Recent evidence indicates that skeletal muscle functions as an endocrine organ by releasing biologically active molecules, termed myokines, during physical exercise. These myokines mediate communication between skeletal muscle and the central nervous system, providing an important biological link between exercise and cognitive health. Among these, irisin, generated through cleavage of fibronectin type III domain-containing protein 5 (FNDC5) (Jin et al., 2018), has received considerable attention because it stimulates hippocampal BDNF expression and promotes synaptic plasticity. Other exercise-responsive myokines, including cathepsin B and meteorin-like protein, have also been implicated in neuronal differentiation, neurogenesis, and memory enhancement (Lee et al., 2021). In addition, exercise-induced lactate functions not only as an energy substrate but also as a signaling molecule capable of stimulating BDNF production and improving neuronal metabolism (Zhu et al., 2025). These observations support the concept of a muscle–brain axis, whereby regular physical activity exerts systemic effects that extend beyond skeletal muscle to preserve cognitive function and delay neurodegenerative processes.

### 5.5 Modulation of neuroinflammation and cytokine responses

Chronic neuroinflammation is increasingly recognized as a key pathological mechanism underlying dementia. Persistent activation of microglia and astrocytes promotes the release of pro-inflammatory cytokines, including tumor necrosis factor-*α* (TNF-α), interleukin (IL)-1β, and IL-6, resulting in synaptic dysfunction, oxidative stress, and progressive neuronal loss (Smith et al., 2012). Regular physical exercise attenuates these inflammatory processes by reducing circulating pro-inflammatory mediators while increasing anti-inflammatory cytokines such as IL-10 (Flynn et al., 2007). Exercise also suppresses oxidative stress by enhancing endogenous antioxidant defense systems, thereby limiting neuronal injury and preserving synaptic integrity (Simioni et al., 2018). Through these combined anti-inflammatory and antioxidant actions, exercise creates a favorable cerebral microenvironment that supports neuronal survival and cognitive function in individuals with dementia.

### 5.6 Vascular and metabolic mechanisms

In addition to its direct effects on neuronal function, exercise improves cerebrovascular and metabolic health, both of which play critical roles in cognitive aging (Shi et al., 2025). Regular aerobic exercise enhances endothelial function, increases cerebral blood flow, and improves vascular reactivity, thereby facilitating oxygen and nutrient delivery to vulnerable brain regions (Tarumi et al., 2025). Improved cerebral perfusion may reduce ischemic injury and contribute to the preservation of cognitive function, particularly in individuals with vascular dementia. Exercise also enhances insulin sensitivity, improves glucose utilization, reduces central adiposity, and favorably modifies lipid metabolism, thereby reducing several established risk factors for dementia (Thyfault and Bergouignan, 2020; Zhang et al., 2024). Furthermore, regular physical activity stimulates mitochondrial biogenesis and reduces oxidative stress, promoting efficient neuronal energy metabolism and decreasing apoptosis (Clemente-Suárez et al., 2024). These vascular and metabolic adaptations collectively contribute to improved brain resilience and may partly explain the protective effects of exercise against cognitive decline.

## 6 Influence of exercise on different dementia types

Dementia comprises a heterogeneous group of neurodegenerative disorders with distinct pathological mechanisms, clinical manifestations, and progression patterns. Consequently, the response to exercise interventions may vary depending on the underlying subtype of dementia. Although evidence is strongest for Alzheimer’s disease and vascular dementia, emerging studies suggest that regular physical activity may also provide functional and cognitive benefits in other forms of dementia (Du et al., 2018). The mechanisms through which exercise exerts these effects differ according to disease pathology, highlighting the importance of individualized exercise prescriptions.

### 6.1 Alzheimer’s disease

Alzheimer’s disease (AD), the most common form of dementia, is characterized by extracellular amyloid-*β* plaque deposition, intracellular neurofibrillary tangles composed of hyperphosphorylated tau protein, synaptic dysfunction, and progressive neuronal loss, particularly within the hippocampus. Regular physical exercise has been shown to improve executive function, attention, and global cognitive performance in individuals with mild to moderate AD (Tenchov et al., 2024). These benefits are thought to arise through enhanced neuroplasticity, increased BDNF expression, improved cerebral perfusion, and attenuation of neuroinflammation. Aerobic exercise has demonstrated the strongest evidence for improving cognitive outcomes, while resistance and multicomponent training contribute to maintaining physical function, muscle strength, and independence in activities of daily living. Although exercise does not halt disease progression, it may delay functional decline and improve quality of life when implemented during the early stages of the disease (Romero Garavito et al., 2025; Yang et al., 2026).

### 6.2 Vascular dementia

Vascular dementia results from cerebrovascular disease leading to reduced cerebral blood flow, chronic ischemia, and vascular injury. Because vascular risk factors such as hypertension, diabetes, obesity, and dyslipidaemia contribute substantially to disease development, exercise may exert particularly pronounced benefits in this subtype (Yang, 2025). Regular aerobic exercise improves endothelial function, cerebral perfusion, blood pressure control, glucose metabolism, and insulin sensitivity while reducing systemic inflammation. These improvements may help slow vascular injury and preserve cognitive function. Resistance training further enhances mobility and functional capacity, whereas multicomponent exercise programs improve balance and reduce fall risk. Consequently, exercise is considered an important adjunctive strategy for both the prevention and management of vascular dementia (Bliss et al., 2021; Schneider et al., 2025).

### 6.3 Dementia with Lewy bodies

Dementia with Lewy bodies (DLB) is characterized by widespread accumulation of *α*-synuclein-containing Lewy bodies and presents with fluctuating cognition, visual hallucinations, parkinsonism, and autonomic dysfunction. Compared with Alzheimer’s disease, evidence regarding exercise interventions in DLB remains limited. Nevertheless, studies suggest that supervised aerobic exercise, balance training, and flexibility exercises may improve gait stability, mobility, postural control, and overall physical function (Liang et al., 2025). These interventions may also reduce fall risk and improve quality of life, although their effects on cognitive outcomes remain uncertain. Because individuals with DLB often experience autonomic instability and motor impairment, exercise programs should be individualized and carefully supervised to ensure safety.

### 6.4 Frontotemporal dementia

Frontotemporal dementia (FTD) is characterized by progressive degeneration of the frontal and temporal lobes, resulting in behavioral changes, impaired executive function, language disturbances, and personality alterations (Neylan and Miller, 2023). Unlike Alzheimer’s disease, memory impairment is often less prominent during the early stages. Evidence supporting exercise interventions in FTD remains scarce; however, regular physical activity may help preserve mobility, improve cardiovascular fitness, reduce behavioral symptoms, and maintain functional independence (De la Rosa et al., 2020). Exercise may also alleviate caregiver burden by improving mood, reducing agitation, and promoting better sleep quality. Given the limited evidence, further randomized controlled trials are required to determine the optimal type and intensity of exercise for individuals with FTD.

### 6.5 Mixed dementia

Mixed dementia, which commonly combines Alzheimer’s pathology with cerebrovascular disease, is increasingly recognized in older adults (Rockwood, 2003). Because both neurodegenerative and vascular mechanisms contribute to disease progression, individuals with mixed dementia may derive benefits from interventions targeting both pathways (Sarhan et al., 2025). Multicomponent exercise programs incorporating aerobic, resistance, balance, and flexibility training appear particularly promising, as they simultaneously improve cardiovascular health, metabolic function, muscle strength, balance, and cognitive performance. Although direct evidence remains limited, current findings suggest that exercise should be considered an important component of comprehensive management for individuals with mixed dementia (Poli et al., 2024; Chen et al., 2025).

## 7 Clinical evidence and exercise prescription in dementia

While the benefits of physical activity in dementia are increasingly recognized, translating this evidence into clinical practice requires careful consideration of how exercise is prescribed. Across studies, considerable variation exists in the type, intensity, and duration of interventions, which makes it challenging to define a single optimal approach. Most randomized trials have employed moderate-intensity aerobic exercise, typically performed three to five times per week, often combined with resistance or balance training; consistent with this, Liu et al. (2020) demonstrated that both aerobic and strength training significantly improved activities of daily living and cognitive function in elderly patients with dementia, although no significant difference was observed between exercise modalities. Programs lasting at least 12–24 weeks appear more likely to demonstrate measurable benefits, as reflected in multidomain interventions such as the Body Brain Life trial (Anstey et al., 2013), although shorter interventions may still yield modest improvements in risk profiles and health behaviors. Importantly, multicomponent interventions that combine aerobic, strength, and balance exercises tend to yield more consistent outcomes compared to single-modality approaches. From a practical standpoint, exercise prescriptions in individuals with dementia should be individualized, taking into account disease severity, comorbidities, baseline functional status, and patient preferences (Mullers et al., 2019). In early stages, individuals may be able to participate in structured and supervised exercise programs, whereas in more advanced stages, simpler and more supported activities may be more appropriate. Supervision is often a key factor influencing both safety and adherence. Interventions delivered in group settings or with caregiver involvement may enhance engagement and sustainability. At the same time, overly intensive or complex programs may reduce compliance, highlighting the need for a balanced and patient-centered approach. Despite growing evidence, standardized guidelines specific to dementia populations remain limited. As a result, clinicians often rely on general physical activity recommendations, adapting them pragmatically to individual needs. Further work is required to establish clear, evidence-based exercise prescriptions tailored to different stages of cognitive decline.

## 8 Heterogeneity in evidence and methodological considerations

One of the recurring challenges in interpreting the literature on exercise and dementia is the considerable heterogeneity across studies (Erickson et al., 2023). Differences in study design, participant characteristics, and intervention protocols make it difficult to draw direct comparisons or establish definitive conclusions.

Variability exists in several aspects, including the type of exercise performed, its intensity and duration, and the stage of dementia among participants. In addition, outcome measures are not always consistent, with some studies focusing on global cognitive scores while others assess specific domains such as memory or executive function. This lack of standardization contributes to mixed findings, particularly in relation to cognitive outcomes. Adherence is another important factor that is often underreported but can significantly influence results (Di Lorito et al., 2020). Individuals who are able to engage consistently in exercise programs are more likely to demonstrate benefit, yet maintaining participation can be challenging in this population. Recognizing these methodological limitations is important when interpreting existing evidence and highlights the need for more standardized and well-designed trials.

## 9 Public health and implementation perspectives

From a broader perspective, the integration of physical activity into dementia care has important public health implications. As the global burden of dementia continues to rise, particularly in low- and middle-income countries, there is an urgent need for interventions that are both effective and scalable (Alshahrani et al., 2024). Exercise-based interventions are relatively low-cost and can be adapted to a variety of settings, including community centers, primary care systems, and even home-based environments. This makes them particularly attractive in resource-constrained settings (Garrett et al., 2011). However, successful implementation requires more than just evidence of efficacy. Factors such as safety, accessibility, cultural acceptability, and availability of trained personnel play a crucial role.

Community-based programs and caregiver-supported interventions may offer practical solutions to improve reach and adherence (Mittelman and Bartels, 2014). In addition, integrating physical activity into existing healthcare frameworks could help bridge the gap between research and real-world practice.

## 10 Conclusion

Physical activity and structured exercise offer a practical and accessible approach to improving outcomes in individuals with dementia. While cognitive benefits are generally modest, consistent improvements are seen in functional ability, mobility, and psychological well-being, all of which are central to maintaining quality of life. These effects likely arise from a combination of neurobiological, vascular, and metabolic mechanisms, with additional relevance in individuals with coexisting conditions such as diabetes. At the same time, variations in study design and challenges related to adherence and implementation highlight the need for more standardized and sustainable approaches. Despite these limitations, increasing physical activity remains a low-cost, adaptable intervention that can be integrated into routine care, and with further refinement, has the potential to play a meaningful role in the prevention and management of dementia.
